# New Intermittent Urinary Micro-Hole Zone Catheter Shows Enhanced Performance in Emptying the Bladder: A Randomised, Controlled Crossover Study

**DOI:** 10.3390/jcm12165266

**Published:** 2023-08-13

**Authors:** Malene Hornbak Landauro, Lotte Jacobsen, Fabio Tentor, Troels Pedersen, Cecilie Rovsing, Omar Feix do Nascimento, Michael Kennelly

**Affiliations:** 1 Coloplast A/S, 3050 Humlebæk, Denmark; dklja@coloplast.com (L.J.); dkfte@coloplast.com (F.T.); dktrope@coloplast.com (T.P.); dkomar@coloplast.com (O.F.d.N.); 2 Sanos Clinic, 9362 Gandrup, Denmark; cec@sanosclinic.com; 3 Department of Urology, Carolinas Medical Center, Charlotte, NC 28207, USA; michael.kennelly@atriumhealth.org

**Keywords:** intermittent urethral catheterisation, urinary bladder disease, urinary flow-stop, micro-hole drainage zone, urinary retention, urinary tract infection

## Abstract

Urinary tract infections (UTIs) are common and troublesome complications of clean intermittent catheterisation (CIC) in individuals suffering from incomplete bladder emptying, which may exacerbate the underlying disease and lead to hospitalisation. Aside from the design of the intermittent catheter and its handling, a recent review highlighted residual urine as one of several UTI risk factors. A new urinary intermittent catheter with multiple micro-holes has been developed for improved bladder emptying. In a controlled crossover study, adult male CIC users were randomised for a health care professional-led catheterisation with the new micro-hole zone catheter (MHZC) and a conventional eyelet catheter (CEC) in two individual test visits to compare the number of flow-stops and the residual urine at the first flow-stop as co-primary endpoints. In 42 male CIC users, the MHZC resulted in significantly fewer flow-stop episodes compared to the CEC (mean 0.17, 95% CI [0.06, 0.45] vs. mean 1.09, 95% CI [0.75, 1.6], respectively; *p* < 0.001) and significantly less residual urine at the first flow-stop (mean 5.10 mL, SE [1.14] vs. mean 39.40 mL, SE [9.65], respectively; *p* < 0.001). No adverse events were observed in this study. The results confirm the enhanced performance of the MHZC compared to a CEC, ensuring an uninterrupted free urine flow with no need to reposition the catheter until the bladder is thoroughly empty.

## 1. Introduction

Urinary tract infections (UTIs) are a common complication of clean intermittent catheterisation (CIC), which impact individuals’ quality of life (QoL) negatively and lead to high health care expenditures for society [1,2,3]. Significantly higher UTI incidence rates and the need for hospitalisation due to UTIs have been observed in CIC users compared to the general population [4]. In a recent review on UTI risk factors, residual urine was identified as one of several risk factors possibly pertaining to the product design of the intermittent catheter used, incorrect handling of the catheter, and a lack of compliance to treatment [5].

**Figure 1:**
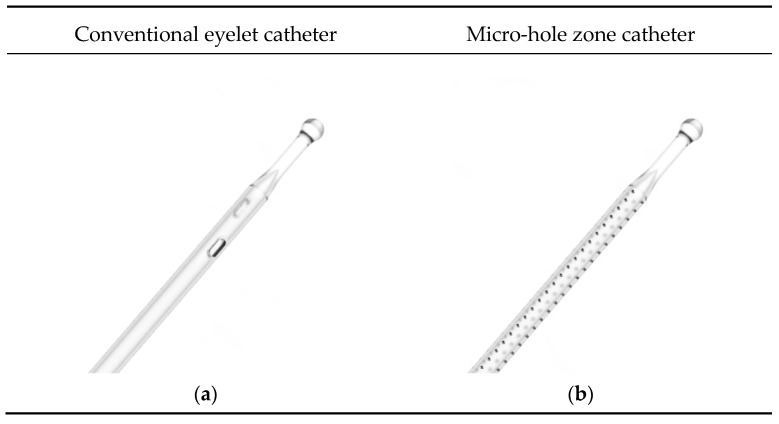
Two different types of flexible intermittent urinary catheters and their drainage zones: (**a**) a conventional eyelet catheter, with two eyelets at the catheter tip; (**b**) the micro-hole zone catheter, with 120 micro-holes extending from the tip down the tube of the catheter, creating a long drainage zone.

To address incomplete bladder emptying, a new urinary intermittent catheter has been developed that features a drainage zone with multiple micro-holes from the tip and extending down the tube of the catheter, as opposed to the conventional eyelet catheters (CEC) available on the market today, which typically feature two drainage eyelets by the catheter tip. The new catheter is herein defined as a micro-hole zone catheter (MHZC) and is depicted side-by-side with a CEC in Figure 1.

One disadvantage of conventional urinary catheters is the need to adjust or reposition the catheter to secure bladder emptying, as advised by nursing guidelines [6,7] and instructions for the use of currently available CECs [8,9,10]. Urinary flow-stops are a consequence of the blockage of the catheter eyelets, which have been described as mucosal suctions, primarily identified with indwelling catheters, which may cause epithelial and vascular changes of the urothelium [11,12,13,14]. Recently, visualisations around the catheter eyelets of intermittent conventional eyelet catheters (from the intraluminal area and the outer catheter surface) have also revealed how the bladder mucosa is sucked into the eyelets during emptying, causing blockages that require the catheter to be repositioned in order to relieve the suction and reinitiate urinary flow [15]. CECs, therefore, carry a similar risk for mucosal suction and flow-stops as indwelling catheters, potentially increasing the infection liability due to the risk of incomplete bladder emptying and iatrogenic microtrauma [5].

The new MHZC, which features a 60-mm-long drainage zone of 80 to 144 micro-holes (depending on its Fr/CH size), ensures a free urine flow and a reduced intra-catheter pressure. Hence, the drainage is largely uninterrupted, ensuring bladder emptying independent of the user technique. As mucosal suctions are prevented, catheter repositioning becomes superfluous and the risk of mucosal microtrauma is averted.

The objective of the current study was to demonstrate the performance of the MHZC compared to the CEC in an out-patient clinical setting. The performance was evaluated in terms of the residual volume at the first flow-stop (RV1), the number of flow-stops, and the intra-catheter pressure. Notably, RV1 represents residual urine in the case where catheterisation is performed without catheter repositioning, which is unnecessary with a MHZC but represents a lack of treatment compliance when using a CEC.

## 2. Materials and Methods

The investigation was a single-centre, randomised, controlled cross-over study performed at Sanos Clinic, Gandrup, Denmark, in the period from August 2022 to November 2022. The study was conducted in accordance with the Declaration of Helsinki II (1964, as amended in Fortaleza, Brazil, October 2013), approved by the Medical Research Ethics Committees (De Videnskabsetiske Medicinske Komitéer case No.2209503), and registered at ClinicalTrials.gov (NCT05485922). All subjects gave their oral and written informed consent before being enrolled in the study.

### 2.1. Study Population

Adult males (>18 years) were included if they had used CIC (size Fr/CH 12 or 14) as their primary bladder emptying method for at least one month, were willing and able to follow study procedures, and had given written consent and signed a letter of authority. Additionally, the subjects had no symptoms of a UTI at the time of inclusion, no allergies to the ingredients in the devices, and had not participated in any other clinical studies during the investigational period (Table 1). 

### 2.2. Study Intervention and Procedure

The study consisted of one inclusion visit and two single test visits during which a catheterisation was performed by a health care professional (HCP) only. The subjects were randomly assigned to two intervention sequences in block sizes of four. The randomisation was centralised using the web-based iMedidata RAVE RTSMTM. Hence, the first group was catheterised with the test device (i.e., the MHZC) at visit 1, and then with a comparator device (i.e., the CEC) at visit 2. The second group tested the devices in reversed order.

Both the test and comparator devices were single-use, sterile, ready-to-use, hydrophilic-coated, flexible, and sleeved catheters. The MHZC had a drainage zone with 120 micro-holes, each with a surface area of 0.13 mm^2^, amounting to a total surface area of ~15 mm^2^. The comparator (CEC) was the commercially available Hollister Vapro^®^ with two oval eyelets, each with a surface area of ~5 mm^2^, amounting to a total surface area of ~10 mm^2^. 

For each of the two test visits, the subjects were requested to show up with a full bladder. The triplicate pre-catheterisation volume was measured with an ultrasound bladder scanner (BladderScan i10™, Verathon, Bothell, WA, USA) and the investigation was initiated only after a pre-catheterisation volume of at least 150 mL was confirmed in all three measurements. Likewise, each test visit was finalised with a triplicate bladder scanner measurement of residual urine post-catheterisation.

**Figure 2:**
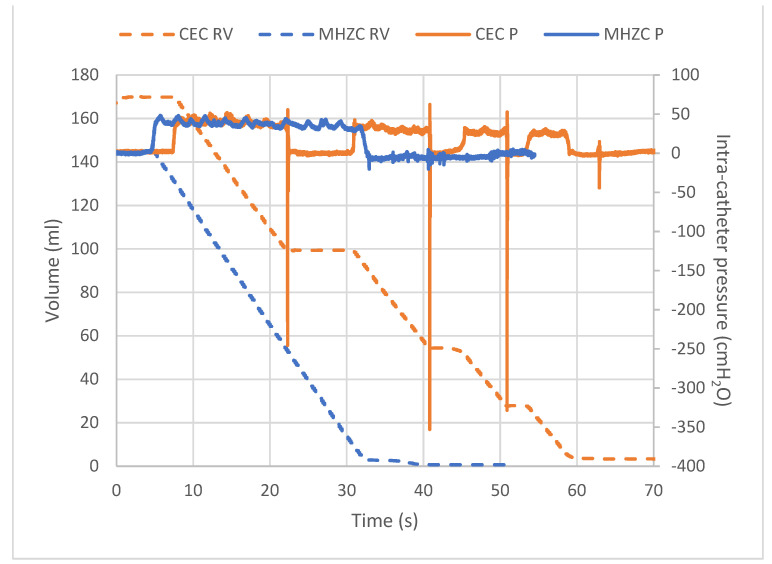
Example of the catheterisation profiles from one participant with the drained urine (mL) and intra-catheter pressure * (cmH2O) as a function of time (seconds) for the micro-hole zone catheter (MHZC, blue) and conventional eyelet catheter (CEC, orange). The solid line represents the intra-catheter pressure (P) measured with a fibre optic sensor and the dotted line represents the drained urine (RV) (mL). * Corrected for atmospheric pressure.

For each catheterisation, the amount of drained urine was measured on a precision weighing scale (A&D, Tokyo, Japan) and the intensity of the flow-stops was measured as the intra-catheter pressure with a fibre optic sensor (FISO Technologies Inc., Quebec, QB, Canada). The lab equipment (Teknologi and Produkt Udvikling A/S, Birkerød, Denmark) can normally measure the pressure and flow in the urethra and bladder, which was adapted by Coloplast A/S so that the sensor was connected with a catheter. The system simultaneously time logged the data at a sampling rate of ~2.5 Hz and generated pressure–volume profiles from where the flowrate, flow-stops, urine volumes, and intra-catheter pressure peaks could be derived. Figure 2 exemplifies the catheterisation profiles of one participant.

Flow-stop episodes were determined as instances where the flowrate decreased to less than 0.8 mL/s for a period of at least two seconds. All episodes were detected by automatic thresholding, followed by manual inspection, where the intra-catheter pressure readings were used to support the assessment of a flow-stop. The last flow-stop was treated as the end of catheterisation and discounted from the total number of flow-stops. Hence, catheterisations with a single flow-stop episode were reported as having no flow-stops.

**Figure 3:**
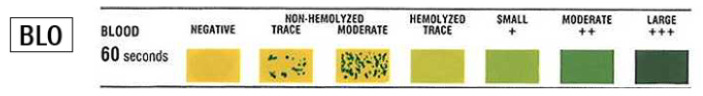
Haematuria assessment from the MultiStix 10 SG Urinalysis Test Strips.

Haematuria in the catheterised urine was assessed with a dipstick test (Siemens Multistix 10 sg) according to a colour scheme (Figure 3) and categorised into a binary variable as either positive (haemolysed 25 Ery/μL(1+), haemolysed 80 Ery/μL(2+), non-haemolysed 80 Ery/μL (2+) and haemolysed 200 Ery/μL(3+)) or negative (negative, non-haemolysed 10 Ery/μL(+/−), haemolysed 10 Ery/μL(+/−)). 

### 2.3. Study Outcomes

The co-primary study endpoints were the number of flow-stop episodes and residual urine volume at the first flow-stop (RV1). RV1 was calculated as the difference between the total volume catheterised (V_Total_) minus the volume catheterised at the first flow-stop (V_1st flow-stop_). Additionally, the residual urine post-catheterisation and intra-catheter pressure at the first flow-stop were included as exploratory endpoints, and haematuria was included as an assessment. Haematuria served as a marker for microtrauma.

### 2.4. Statistical Methods

The sample size calculation was based on two previous exploratory studies (available at ClinicalTrials.gov: NCT04445051 and NCT04543136). Taking a discontinuation rate of 20% into consideration, both primary endpoints were sufficiently supported with 90% power by randomising at least 42 subjects. 

The two co-primary endpoints, RV1 and flow-stop episodes, were analysed using a linear mixed model and a generalised linear mixed model, respectively, with the participants as a random component and visits (visit 1 and 2) and devices (comparator and test device) as fixed effects, assuming statistical significance at *p* < 0.05. The pass criteria for the study were based on the results from analysing the two primary endpoints in a hierarchical fashion, rejecting the null-hypothesis on the first endpoint (residual urine at the first flow-stop) before continuing to the second (number of flow-stop episodes). The robustness of the primary analysis of RV1 was further confirmed by a Wilcoxon signed rank test to evaluate the assumption of normality.

The exploratory endpoints (intra-catheter pressure and residual volume post-catheterisation) were analysed with a generalised linear mixed model as the primary endpoint. In a post hoc analysis, a positive haematuria assessment was analysed using a generalised linear mixed model, modelling the probability (odds ratio—OR) of a positive outcome in favour of the MHZC. 

All analyses were conducted using SAS statistical software (SAS Institute Inc., Cary, NC, USA, version 6.4/Enterprise Guide version 7.1).

## 3. Results

**Figure 4:**
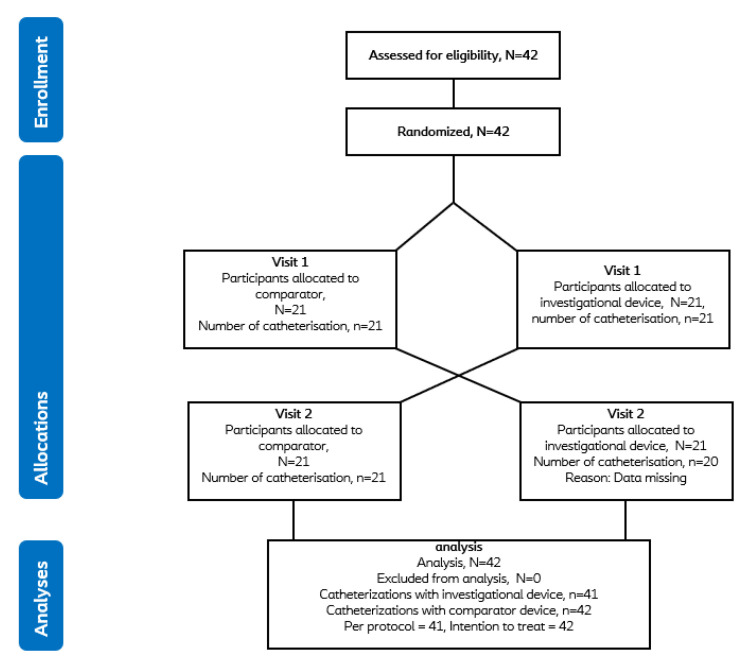
Flow diagram of subjects randomised into two different intervention sequence groups.

Forty-two male CIC users were screened for eligibility, and all met the inclusion criteria and were enrolled and randomised from August to November 2022. All but one subject completed all visits, with data missing for one subject during visit 2. Accordingly, 41 catheterisation profiles were recorded for the investigational device and 42 for the comparator (Figure 4).

The baseline characteristics are presented in Table 2. The mean age was 68 (range: 41–87 years) with a 3:2 split between individuals with non-neurogenic and neurogenic bladder dysfunctions. Benign prostatic hyperplasia was the predominant cause of CIC use (62%), followed by 21% with spinal cord injury, 10% with multiple sclerosis, 5% with strictures, and 2% with prostate cancer. No adverse events were reported.

### 3.1. Residual Urine at the First Flow-Stop

**Figure 5:**
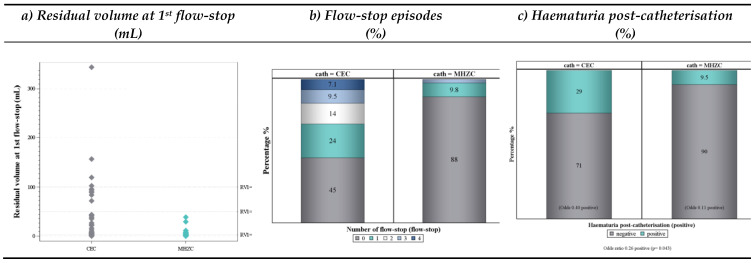
Frequency plots for the residual volume at the first flow-stop, flow-stop episodes, and haematuria after catheterisation with both the MHZC and CEC: (**a**) a scatter plot depicting the distribution of the residual volume (mL) at the first flow-stop upon catheterisations with the CEC (grey) and MHZC (turquoise) and horizontal lines at 10 mL, 50 mL, and 100 mL; (**b**) number of flow-stop episodes during catheterisations with the CEC and MHZC categorised into 0–4 episodes (0 flow-stops = grey; 1 flow-stops = turquoise; 2 flow-stops = white; 3 flow-stops = light blue; 4 flow-stops = dark blue); (**c**) proportions of positive (turquoise) and negative (grey) haematuria post-catheterisation measured with a dipstick after catheterisations with the CEC and MHZC. MHZC = micro-hole zone catheter; CEC = conventional eyelet catheter.

The mean residual urine at the first flow-stop (95% CI) was significantly lower for the MHZC with 5.10 mL [2.79; 7.42] compared to 39.40 mL [19.92; 58.89] for the CEC, with a mean difference of 34.30 mL [14.69, 53.91] at *p* < 0.001 (Table 3). Most catheterisations with the MHZC (90%) had RV1 values below 10 mL, and none had RV1 values above 50 mL. For the CEC, 52% of the catheterisations had RV1 values below 10 mL, 24% above 50 mL, and 10% above 100 mL (Figure 5a).

### 3.2. Flow-Stop Episodes

Catheterisation with the MHZC resulted in close to zero flow-stops, with a mean (95% CI) of 0.17 [0.06, 0.45], compared to an average of one flow-stop with the CEC and a mean of 1.09 [0.75, 1.6]. Only 4 out of 41 catheterisations with the MHZC (10%) had flow-stops, of which three led to one flow-stop and one led to three flow-stops. Conversely, more than half of the catheterisations with the CEC had at least one flow-stop and more than 30% of the catheterisations had at least two flow-stops. Hence, flow-stops were 84% less likely to happen with the MHZC compared to the CEC (*p* < 0.001) (Table 3 and Figure 5b).

### 3.3. Intra-Catheter Peak Pressure

The intra-catheter pressure was measured over a range of −87.7 to 0 cmH2O for the MHZC and a range of −423.2 to 0 cmH2O for the CEC. The mean suction pressure at the first flow-stop (95% CI) was −16.5 cmH2O [−22.9; −10.0] for the MHZC versus −113.0 cmH2O [−156.4; −71.7] for the CEC, with a mean difference of 97.6 cmH2O [−140.4, −54.8], which was statistically significant at *p* < 0.001 (Table 3). 

### 3.4. Haematuria

The proportions of catheterised urine with a positive dipstick haematuria were 10% for the MHZC compared to 29% for the CEC. Hence, haematuria was 74% less likely with the MHZC compared to the CEC (*p* < 0.05) (Table 3 and Figure 5c).

### 3.5. Post-Catheterisation Volume (Bladder Scanner)

The residual urine post-catheterisation was assessed with an ultrasound bladder scanner, and the mean values (CI 95%) were low and similar for both catheters at 5.92 mL [−2.17, 14.01] for the MHZC compared to 7.13 mL [−2.39, 16.64] for the CEC, with a non-significant difference (*p* = 0.846) (Table 3).

### 3.6. Safety

No adverse events were observed during this study.

## 4. Discussion

The study results demonstrated the superior performance of the micro-hole zone catheter (MHZC) in male CIC users through a significantly reduced number of flow-stop episodes and significantly reduced residual volume at the first flow-stop compared to a conventional eyelet catheter (CEC). This was substantiated by significantly smaller pressure peaks at the first flow-stop and further corroborated by significantly less instances of haematuria upon catheterisation with the MHZC compared to the CEC.

Despite advances in intermittent catheterisation for individuals with incomplete bladder emptying, UTIs in CIC-dependent users remain a cause of concern. These UTIs can be resource-demanding, highly distressing, and impact QoL, especially when the underlying disease is affected [1,2,16,17,18,19]. While catheterising 4 to 6 times per day to mimic normal bladder filling and emptying is a crucial element of the CIC regimen, the catheterisation technique with the actual intermittent catheter is just as important. The catheterisation procedure consists of numerous steps, stressing the need for support and proper instruction to ensure the correct catheterisation technique and reduce the risk of complications.

The UTI risk factor model [5] describes a number of catheter-related risks, including a lack of proper hygiene introducing bacteria upon insertion [20,21], residual urine [22,23,24], and microtrauma to the urethra or bladder mucosa caused by repeated catheterisation [25,26,27]. The new catheter has been designed to address these risk factors, with a drainage zone featuring multiple micro-holes. Compared with conventional eyelet catheters, these micro-holes enable enhanced bladder emptying by reducing the hydrodynamic pressure around each micro-hole, thereby bypassing the risk of mucosal suction [15]. 

In the current study, the residual urine at the first flow-stop was significantly lower upon catheterisations with the MHZC compared to the CEC, securing bladder emptying to less than 10 mL at the first flow-stop occurrence in nine out of ten catheterisations. As this endpoint represents residual urine in the case where catheterisation is performed without adjusting the catheter upon flow-stops, it serves as an example of the consequence of premature catheter removal with the CEC. 

The association between residual urine and the UTI risk has been thoroughly investigated, although most studies involve individuals who void without a catheter or involve mixed populations of both CIC users and non-users or with different medical histories [28,29,30,31,32,33,34]. Adding to the complexity, the definition of UTIs often varies across trials, rendering the task to assess and generalise the results within this area challenging [35]. 

Nonetheless, the clinical implication for residual urine relates to urine as a potential growth medium for certain persistent bacterial strains such as *E. coli*, despite urine’s natural antimicrobial properties [36]. Hence, significantly higher bacterial loads (≥10^6^ CFU/mL) have been observed in those with abnormal post-void residuals compared to patients with lower volumes (*p* < 0.001) [37]. Additionally, a greater rate of UTIs (measured as positive bacteriuria) has been observed with increasing residual urine volumes among neurogenic CIC users compared to patients with low volumes [24] and compared to patients on prophylactic antibacterial treatment [32]. Since long-term antibacterial treatment is contraindicated due to a risk of developing bacterial resistance, efficient bladder emptying should be an important focus area for CIC users and HCPs as a means to reduce one of many UTI risk factors. Interestingly, a recent CIC user survey revealed that subjects with an increased UTI risk were more likely to perceive an inability to thoroughly empty their bladder, defined as the residual urine volumes after performing a CIC [18]. Thus, CIC users’ perceptions of symptoms and patient-reported outcomes are important to acknowledge and can potentially aid in identifying issues concerning a lack of proper CIC technique or future UTI risks factors to be addressed [38]. 

As such, the MHZC supports CIC users’ adherence to guidelines that recommend CIC users to ensure thorough bladder emptying because drainage through this catheter is associated with bladder emptying without premature flow-stops. 

The clinical implication of flow-stops and associated mucosal suctions observed in the CEC concerns the effect of repositioning the catheter to release the bladder wall from catheter eyelets. Endoscopic visualisation during catheterisation with three types of CEC in a pre-clinical porcine bladder model has revealed scraping of the mucosa layer, which left tissue residues on the edges of the eyelets and increased the turbidity and floating tissue agglomerates in the drained liquid [15]. In support of this, mucosal oedemas and injuries leading to occasional minor haemorrhages have also been observed in several studies in association with catheter repositioning with indwelling catheters from both pre-clinical and clinical settings [11,12,14,39].

In the current study, there was a 74% higher likelihood for haematuria with the CEC compared to catheterisations with the MHZC, which was supported by a significant reduction in mucosal suction pressure. This pressure peak reduction can be directly linked to the decreases in eyelet size and the total number of eyelets. As the size of the drainage holes of the MHZC is ten times smaller than conventional eyelets and the micro-holes extend along the catheter covering a long drainage zone, the risk of mucosal suction was significantly reduced by 84%. Consequently, continuous daily catheterisations with a CEC 4–6 times a day and an 84% higher risk for iatrogenic micro-trauma confront the CIC-dependent user with a significant risk on a yearly basis, potentially inflicting increased intervention and health-care costs.

Another consequence of these mucosal lesions is a potential compromise of the host defence mechanism against bacteria, which may serve as a habitat for adhesive bacteria [40]. Hence, using a ventilated catheter compared to a non-ventilated system resulted in a significantly reduced rate of bacteriuria in female patients because the atmospheric pressure was maintained in balance with a ventilation hole [41]. In the already susceptible patient on a CIC, suction-associated mucosal microtrauma may further affect the infection liability if the natural barrier function is continuously injured [42,43]. Nonetheless, the results from the current study present possible risk factors associated with intermittent catheterisations. Any direct associations between the design of the drainage zone of urinary catheters and the risk for future UTIs require a different study design where information on the UTI rate is gathered long-term upon self-led catheterisation in a home setting.

The strength of this study regards the cross-over design, where each participant acts as their own control, and the statistical significance of the two co-primary endpoints. Hence, it can be concluded that the enhanced performance of the MHZC is associated with a true and robust result. Additionally, the co-primary endpoints are in line with results from two previous exploratory studies performed in healthy volunteers and in male CIC users (NCT04445051 and NCT04543136). The study limitations include the HCP-led catheterisations in a hospital setting from two hospital visits only, meaning the study does not simulate long-term self-catheterisation in a real-world setting. However, performance endpoints such as the flow-stop episodes, residual urine at the first flow-stop, and intra-catheter peak pressure would not be possible to measure in a home-setting. Additionally, differences pertaining to underlying disease (e.g., neurological vs. non-neurological disease states) have not been investigated, as the current study endpoints only concerned mode of action differences between conventional eyelet catheters and micro-hole zone catheters.

Another study limitation concerns the origin of haematuria, which can only be theoretically linked to the bladder wall as a result of the associated intra-catheter peak pressure. The patient’s medical history and potentially difficult and traumatic catheterisations may also lead to haematuria, which can be observed for individuals with urethral stenosis and benign prostatic hyperplasia, with the latter being due to increased hypervascularisation of the prostate [44]. However, the patient’s medical history was included as a variable in the statistical analyses and accounted for.

The observed pressure peaks with a mean of ~−100 cmH2O were slightly smaller compared to the pressure peaks observed in the pre-clinical setting. The type of equipment, eyelet and lumen sizes, catheter length, abdominal pressure, and catheter material are possible explanations for this difference [11,15]. Nonetheless, the minimum peak pressure value at the first flow-stop for the CEC in the current study was as low as –423 cmH2O and even greater peaks (lower values) were observed at the first flow-stop. As the iatrogenic microtrauma is likely related to both oedema during suction through the eyelets and tearing and scraping of the mucosal tissue by the eyelets during catheter adjustment, greater pressure peaks are expected to injure more [15].

Finally, the generalisability of the current study results can be discussed due to a potential selection bias for the clinical study participants related to the inclusion and exclusion criteria. Nonetheless, the co-primary endpoints are considered independent of the disease state, confirmed by the generalisability of the results between two exploratory studies performed in healthy volunteers and CIC users, respectively, and the current study.

## 5. Conclusions

The results from the current study confirm the superior performance of the micro-hole zone catheter compared to the CEC. In an already susceptible group of individuals, the MHZC, thus, provides CIC users with a new generation of catheters securing complete bladder emptying in an uninterrupted free flow with no need for repositioning, potentially minimising the risk for UTIs.

## Figures and Tables

**Table 1:** Study inclusion and exclusion criteria.

Inclusion Criteria:	Exclusion Criteria:
Male	Participation in any other clinical study during the investigation
Was at least 18 years of age and had full legal capacity	Symptoms of urinary tract infection as judged by the investigator
Had given written informed consent and signed a letter of authority	Any known allergies to ingredients in the products
Had used clean intermittent catheterisation CH12 or CH14 for at least one month and used intermittent catheterisation as the primary bladder emptying method	Relevant medical history that would prevent the subject to participate in the investigation (investigators’ judgement)
Was able and willing to follow study procedures	

**Table 2:** Baseline demographics.

Total	N = 42
Age (years), mean (range)	**68.0 (41; 87)**
Non-neurogenic bladder dysfunction, n (% total)	**29 (69)**
Neurogenic bladder dysfunction, n (% total)	**13 (31)**
Medical history	
*Benign Prostate Hyperplasia*	26 (62)
*Spinal Cord Injury*	9 (21)
*Multiple Sclerosis*	4 (10)
*Strictures*	2 (5)
*Prostate Cancer*	1 (2)

**Table 3:** Performance outcomes for the MHZC and CEC and their statistical differences. Performance outcomes were evaluated as the mean number of flow-stops, residual urine at the first flow-stop, suction pressure peak at the first flow-stop, residual urine post-catheterisation, and the probability of a positive haematuria assessment. The statistical analyses tested the hypothesis of differences between the MHZC and CEC.

	Mean ^Ω^ [95% CI]		Mean Difference [95% CI]	Ratio [95% CI]	*p*-Value
	MHZC	CEC			
Flow-stop episodes, number	0.17 [0.06; 0.45]	1.09 [0.75; 1.60]	-	0.16 * [0.05; 0.44]	<0.001
Residual urine at the first flow-stop, mL	5.10 [2.79; 7.42]	39.40 [19.92; 58.89]	34.30 ** [14.69; 53.91]	-	0.001
Suction pressure peak, cmH2O	−16.5 [−22.9; −10.0]	−113.0 [−156.4; −71.7]	−97.6 ** [−140.4; −54.8]	-	<0.001
Residual urine post-catheterisation, mL	5.92 [−2.17; 14.01]	7.13 [−2.39; 16.64]	1.21 ** [−11.10; 13.51]	-	0.846
Probability of a positive haematuria	0.10 [0.03; 0.24]	0.29 [0.17; 0.45]	-	0.26 *** [0.07; 0.96]	0.043

^Ω^ Micro-hole zone catheter (MHZC); conventional eyelet catheter (CEC). * Relative risk ratio measuring the probability in favour of the MHZC, i.e., RR < 1. ** Mean difference between the CEC and MHZC ≠ 0 in favour of the MHZC. *** Odds ratio measuring the odds of a negative assessment in favour of the MHZC, i.e., OR < 1.

## Data Availability

The data presented in this study are available at https://clinicaltrials.gov/ (NCT05485922) and from the corresponding author upon request.
